# Draft Genome Sequence of *Corynebacterium variabile* Mu292, Isolated from Munster, a French Smear-Ripened Cheese

**DOI:** 10.1128/genomeA.00669-16

**Published:** 2016-07-21

**Authors:** Eric Dugat-Bony, Anne-Sophie Sarthou, Valentin Loux, Marie Vidal, Pascal Bonnarme, Françoise Irlinger, Séverine Layec

**Affiliations:** a UMR Génie et Microbiologie des Procédés Alimentaires, GMPA, AgroParisTech, INRA, Université Paris-Saclay, Thiverval-Grignon, France; b MaIAGE, INRA, Université Paris-Saclay, Jouy-en-Josas, France; c INRA, GeT-PlaGe, Genotoul, Castanet-Tolosan, France; d INRA, UAR1209, Castanet-Tolosan, France

## Abstract

Here, we report the draft genome sequence of *Corynebacterium variabile* Mu292, which was originally isolated from the surface of Munster, a French smear-ripened cheese. This genome investigation will improve our knowledge on the molecular determinants potentially involved in the adaptation of this strain during the Munster-type cheese manufacturing process.

## GENOME ANNOUNCEMENT

Smear-ripened cheeses harbor complex microbial consortia that are mainly responsible for the production of typical sensory properties (1). Their activities are influenced by the technological processes and manufacturing environment. *Corynebacterium* species are commonly involved in the cheese-ripening process (2–4) and contribute to the flavor and texture of the final product. Three sequenced genomes are currently available for cheese isolates belonging to the *Corynebacterium* genus. Two are affiliated with *Corynebacterium casei* and were isolated from a French (5) and an Irish smear-ripened cheese (6), respectively. The third one is affiliated with *Corynebacterium variabile* and was isolated from Gubbeen (7).

We report here the genome sequence of *Corynebacterium variabile* Mu292, isolated in 1989 from Munster, a soft smear-ripened cheese. Sequencing was performed using Illumina MiSeq technology. After filtering, a total of 1,169,642 paired-ends reads of 250 bp in length were generated and merged using FLASH (8). *De novo* assembly was performed using SPAdes (version 3.1.1, with default parameters) (9), which generated 66 large contigs (≥1,000 bp), with an average sequencing coverage of 100-fold. The unclosed draft genome is 3,185,550 bp in length and has a G+C content of 67.3%. Gene prediction and annotation were performed using the IMG system, as described previously (10). This genome encompasses 3,007 genes, including 2,942 coding DNA sequences, 7 rRNAs, and 58 tRNAs.

Comparative analysis of the genome of *C. variabile* Mu292 with the genome of *C. variabile* strain DSM 44702, isolated from Gubbeen cheese (7), will provide valuable insights into the adaptation of *C. variabile* strains to different cheese technologies. Indeed, Gubbeen and Munster cheeses are differentiated by their technological characters, such as pH of the curd and NaCl and dry-matter contents (11, 12). Interestingly, the presence of a type I restriction-modification system in the genome of *C. variabile* Mu292 might explain why it is devoid of the phage-related chromosomal island of *C. variabile* DSM 44702 (7, 13).

Another feature in the genome of *C. variabile* Mu292 is the presence of a gene coding for a putative arylsulfatase (EC 3.1.6.1), sharing 82% sequence identity (protein level) with the sequence of *Corynebacterium terpenotabidum* Y-11^T^ (NCBI accession no. WP_020440046), a bacterium isolated from soil and which is phylogenetically close to *C. variabile* (14, 15). This enzyme has been previously described in various soil bacteria and is considered as a key enzyme in sulfur metabolism (16, 17). In the cheese habitat, arylsulfatase may be involved in the release of molecules conjugated with sulfate, such as alkylphenols, which contribute to sheep-like flavors of the cheeses manufactured from sheep’s milk (18). Thus, this specificity found in the genome of *C. variabile* Mu292 might be of interest for understanding sulfur metabolism in cheese, which is of great importance for the cheese-making process (19).

This second genome sequence of *Corynebacterium variabile* will allow deeper comparative genomic studies among *Corynebacterium* species and other *Actinobacteria*, provides new elements for understanding the adaptation strategies of cheese bacteria to the cheese habitat, and potentially aids in discovering novel technological properties for the food industry.

### Nucleotide sequence accession numbers.

The draft genome sequences of *Corynebacterium variabile* Mu292 have been deposited at the EMBL database under accession numbers FAUH01000001 to FAUH01000066.
